# Heat and Mass Transfer Modeling to Predict Temperature Distribution during Potato Frying after Pre-Treatment with Pulsed Electric Field

**DOI:** 10.3390/foods10081679

**Published:** 2021-07-21

**Authors:** Gohar Gholamibozanjani, Sze Ying Leong, Indrawati Oey, Phil Bremer, Patrick Silcock, Mohammed Farid

**Affiliations:** 1 Department of Chemical and Materials Engineering, The University of Auckland, Auckland 1023, New Zealand; ggho641@aucklanduni.ac.nz; 2 Department of Food Science, University of Otago, Dunedin 9016, New Zealand; sze.leong@otago.ac.nz (S.Y.L.); indrawati.oey@otago.ac.nz (I.O.); phil.bremer@otago.ac.nz (P.B.); pat.silcock@otago.ac.nz (P.S.); 3 Riddet Institute, Palmerston North 4442, New Zealand

**Keywords:** potato, frying, PEF, variable space network method, enthalpy method, approximate quasi-steady-state analysis, explicit finite difference

## Abstract

Based on unsteady state heat conduction, a mathematical model has been developed to describe the simultaneous heat and moisture transfer during potato frying. For the first time, the equation was solved using both enthalpy and Variable Space Network (VSN) methods, based on a moving interface defined by the boiling temperature of water in a potato disc during frying. Two separate regions of the potato disc namely fried (crust) and unfried (core), were considered as heat transfer domains. A variable boiling temperature of the water in potato discs was required as an input parameter for the model as the water is evaporated during frying, resulting in an increase in the soluble solid concentration of the potato sample. Pulsed electric field (PEF) pretreatment prior to frying had no significant effect on the measured moisture content, thermal conductivity or frying time compared to potatoes that did not receive a PEF pretreatment. However, a PEF pretreatment at 1.1 kV/cm and 56 kJ/kg reduced the temperature variation in the experimentally measured potato center by up to 30%. The proposed heat and moisture transfer model based on unsteady state heat conduction successfully predicted the experimental measurements, especially when the equation was solved using the enthalpy method.

## 1. Introduction

Deep-oil frying is one of the most common processes used for food preparation, which is estimated as a billion-dollar industry worldwide [1]. Accordingly, monitoring and simulation of the temperature time distribution in food during frying is important to warrant the final quality of the fried food products [2]. Depending on the processing intensity, moisture in foods is removed partially or fully during frying. In fact, a phase change from liquid to vapor happens due to absorbing heat from hot oil during frying. Therefore, in the food frying process, heat and mass transfer phenomena take place simultaneously.

Deep fried potatoes are among the most popular of food products owing to their availability, convenience, price and taste [3]. Several researchers have developed mathematical models to describe the frying process of potatoes [4,5,6]. These models have been developed to describe heat and mass transfer mechanism for potato frying based on either single or two-phase systems. Based on the assumption that heat and mass transfer processes occur independently, an analytical correlation for heat and moisture transfer coefficients based on different potato geometries was developed by Dincer [7], who used a single-phase model by solving the diffusion equation for both the heat and mass transfer phenomena, without coupling the two phases together. Costa and Oliveira [8] developed a two-phase model (crust and core) with a time-dependent boundary condition to define the water loss in fried potato discs. In their model system, dynamics were described as a general first-order response to an exponential input, where the rate constants of both phases (crust and core) were highly dependent on the thickness of potato discs while the rate constant of the unfried phase (core) was impacted by the frying temperature. These researchers reported that 63% of the initial moisture content in a potato still resides in the unfried phase.

Farkas et al. [9] developed a two-phase domain model to describe the heat and mass transfers in food frying, including potato frying. In this model it was assumed that water boiling occurs at a moving interface similar to the process of melting and solidification [10]. To avoid complicated and time-intensive models, some researchers have assumed that the process can be described by heat transfer equations only. For example, Farid and Chen [4] employed the unsteady state heat conduction equation and used the finite difference method of Murray and Landis [11] to solve the equation. Their model eliminated the use of excessive parameters such as diffusion coefficient and mass transfer coefficient (in addition to heat transfer parameters) which are difficult to measure analytically. Their work successfully described the frying process but failed to predict the gradual increase of potato center temperature in the final period of frying. To address this issue, Southern et al. [12] defined the center of the potato disc as a region, rather than a point, based on the cross-section area of the thermocouple used. Then, an integration was carried out within that location. This assumption reduced the errors caused by the displacement of the thermocouple from the center of the potato disc. They also considered a variable surface heat transfer coefficient, which was high during boiling and lower during sensible heating. Lastly, they considered the oil penetration effect, which altered the properties of the fried potato discs and thus slowed down the increase in the center temperature of the potato during the final sensible heating period. However, with this approach, the agreement between predicted and measured temperature was poor during this final heating period.

The use of pulsed electric field (PEF) technology as a pre-treatment in the potato industry, especially for the production of deep-fried potato products (e.g., French fries and potato crisps), has shown promise as a means to: achieve better process performance as the texture of PEF-treated potatoes are more flexible, easier to cut and have a smoother cut surface; reduce energy and water consumption during production as the integration of PEF into existing process line can speed up the production time and reduce energy and water use compared to conventional processing; produce better quality end product as PEF facilitates uniform browning after frying an reduced oil uptake; and minimize food waste, as PEF-treated potatoes have less tissue damage and are less prone to breaking [13,14]. PEF treatment involves applying short (μs or ms in duration) and repetitive electric pulses of high voltage across the potato which is positioned between two conducting electrodes [15]. Electric field strength, pulse frequency, pulse number, pulse shape and polarity, pulse width and duration are important operating parameters of PEF processing [16]. When the electric field strength of the applied pulses exceeds thresholds electrical potential of the cell membrane, existing pores in the membrane can enlarge which affects its microstructure [17]. These changes will also impact heat, mass and momentum transfers, and affect the rate of chemical reactions, moisture content and oil uptake during the subsequent frying process [18]. However, heat and mass transfers during frying of PEF-treated potatoes has not yet been reported.

To accurately model the frying process of potatoes is not trivial, as it is postulated that the water boiling temperature will increase as the boiling interface moves towards the potato center owing to increases in the concentration of solid soluble matters in the potato discs as water is evaporated. To address this, an empirical correlation for water boiling temperature as a function of interface position of fried (crust) and unfried (core) regions was developed in the current study. The unsteady state heat conduction equation was then solved using the enthalpy method, for the first time for potatoes, in addition to the well-known Variable Space Network (VSN) method previously used by other researchers [19]. The enthalpy method or the effective heat capacity method has previously been widely used to describe melting and solidification of materials [10,20] and unlike the VSN method, it has been applied to two- or three-dimensional geometry samples [21,22]. The enthalpy method was applied to help overcome the computation limitations of the VSN method when an explicit finite difference method is used in the solutions. However, a limitation of the enthalpy method is the assumption that water boils within a very narrow range of temperature. Both the enthalpy method and the VSN method were used in this study to predict temperature-time distribution at different locations of the potato disc. The complete frying time was also measured directly using the method developed by Michael and Farid [23] for flat geometries.

Thus, this study aims to test whether the enthalpy and VSN methods based on a moving interface defined by the boiling temperature of water in potato during frying can describe the temperature-time distribution of PEF-treated potatoes.

## 2. Materials and Methods

### 2.1. Sample Preparation and PEF Treatment

All potatoes used in this study were harvested together, graded, washed and stored in jute potato bags at 10 °C until use. Thirty individual potatoes were randomly allocated to either untreated or PEF treated. Potatoes were PEF-treated using the batch mode of an ELCRACK-HVP 5 (German Institute of Food Technologies, Quakenbrueck, Germany) unit by positioning each potato tuber in the middle of the batch treatment chamber (100 mm length × 80 mm width × 50 mm height, 400 mL capacity) consisting of two parallel stainless-steel electrodes of 5 mm thickness separated by a distance of 80 mm. To standardize the transmission of the electric currents, the whole potato tuber was fully submerged in sodium phosphate buffer (10 mM; pH 7) [24]. Potatoes were treated with PEF at an electric field strength of 1.1 kV/cm, pulse frequency of 50 Hz, and pulse width of 20 µs at varying pulse numbers of 1100 and 3100 that resulted in specific energy inputs at either 54.8–57.7 kJ/kg (thereafter referred as “PEF Low”) or 149.2–159.6 kJ/kg (“PEF High”), respectively. During PEF treatment, the pulse shape (square wave bipolar) was monitored on-line using an oscilloscope (Model UT2025C, Uni-Trend Group Ltd., Dongguan, China). The output voltage, output current, pulse energy, pulse number and load resistance was monitored during treatment and the temperature and electrical conductivity of the conducting medium were measured before and after PEF treatment, using a conductivity/temperature meter (CyberScan CON 11, Eutech Instruments, Queenstown, Singapore).

### 2.2. Moisture Content Measurement

To determine their moisture content (*w*), samples were cut from the surface and center of each potato (either untreated or PEF-treated) and placed in a convection oven (Binder drying and heating chambers ED115, Tuttlingen, Germany) at 140 °C for 4 h. Potato mass was measured over time until a constant mass was achieved and the moisture content was calculated for triplicates independent samples.

### 2.3. Thermal Conductivity Measurements

**Figure 1:**
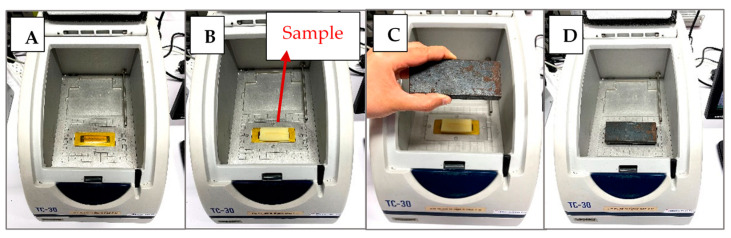
Stepwise potato sample preparation for the thermal conductivity measurement: (**A**) the empty ThermTest TC-30 machine; (**B**) placing sample in the machine; (**C**) a weight added to the sample; and (**D**) when the weight was on the sample.

Thermal conductivity was measured using a ThermTest TC-30 (Fredericton, NB, Canada), which works based on transient heat conduction. Fresh or fried potatoes were cut in rectangular shapes (50 mm length × 25 mm width × 5 mm height) and their thermal conductivity was measured as shown in Figure 1. Briefly, the sample was placed on the spring-loaded sensor (Figure 1A,B) and a weight placed on top of it to ensure proper contact (Figure 1C,D). A heat reflectance sensor was used so that the heating element was supported on an insulated backing and surrounded by a guard ring for one-dimensional heat flow. Heat was generated when a current was applied to the sensor coil. Simultaneously, the rate of temperature increase was monitored by the voltage drop in the sensor, which was calibrated to the temperature change. The thermal conductivity of the sample was inversely proportional to the rate of increase in the temperature. In other words, samples with a lower thermal conductivity showed a steeper rise in temperature. Thermal conductivity was measured in triplicate using independent samples.

### 2.4. Potato Frying Experiment

**Figure 2:**
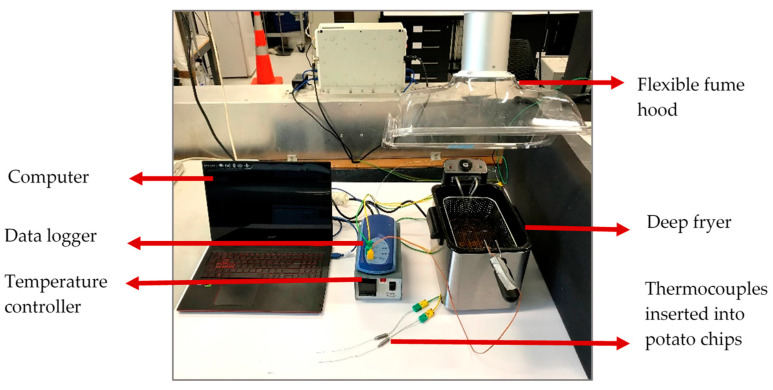
Potato frying experiment setup.

**Figure 3:**
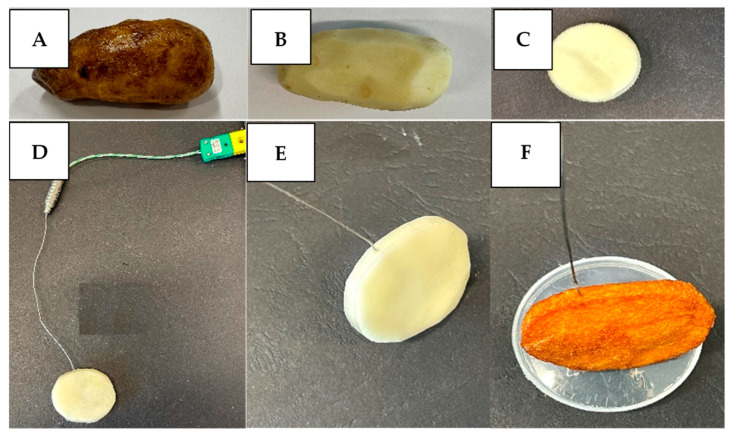
Stepwise sample preparation for the potato frying experiment: (**A**) intact potato after PEF treatment; (**B**) removal of skin; (**C**) cutting sample in a circular disc form; (**D**,**E**) position of thermocouple inserted into the sample before frying; and (**F**) position of thermocouple in the sample upon completion of frying experiment.

Both the PEF-treated and the untreated potatoes were peeled and cut in circular discs (40 mm diameter and 5 mm thickness). A K-type thermocouple (IEC K 0.5 × 150 mm) was inserted into the center of the potato disc to measure its temperature during frying. The potato disc was placed in a 4 L temperature-controlled deep fryer (Brabantia Deep Fryer BBEK1130, Valkenswaard, The Netherlands, 393 × 280 × 280 mm) as shown in Figure 2. The oil was held at 180 °C [25] using a temperature controller. Frying temperatures were recorded over time using PicoLog TC-08 data logger (Cambridgeshire, UK) (Figure 2). The potato preparation including skin removal, cutting and thermocouple insertion in the center of potato discs is shown in Figure 3.

### 2.5. Measurement of Water Boiling Temperature of Potato

The water content of a potato ranges between 70% to 80% [26] and during frying most of it is evaporated when the water temperature reaches “boiling” [27]. However, since the soluble matter in a potato such as sugar and potassium become more concentrated during frying, the water boiling temperature in the potato is expected to increase over time. This temperature elevation is expected to continue until a major proportion of free water is removed from potato and the center temperature reaches the targeted oil temperature (180 °C). To understand the elevation mechanism of water boiling temperature, potato discs were fried in the same fryer as outlined in Section 2.4. As soon as the center temperature of the potato disc started to rise above the water boiling temperature (i.e., >100 °C as monitored using the data logger), the disc was removed immediately from the hot oil. The water content remaining in each partially fried potato disc was determined by placing them in an oven at 140 °C to dry, until a constant weight was achieved, which amount was calculated based on the known initial moisture content of potato (Equations (1)–(3)). Equation (3) represents the moisture content of potato in which water boiling point started to deviate from 100 °C.

Initial water mass (g) = Initial potato mass before drying (g) − Potato mass after drying in an oven (g)(1)

Water remained after partial frying (g) = Potato mass when frying stopped (g) − Potato mass after drying in an oven (g)(2)

(3) $$\%\;\mathrm{Water}\;\mathrm{remaining}\;\mathrm{after}\;\mathrm{partial}\;\mathrm{frying}\;=\frac{\mathrm{Water}\;\mathrm{remained}\;\mathrm{after}\;\mathrm{partial}\;\mathrm{frying}\;(g)}{\mathrm{Initial}\;\mathrm{water}\;\mathrm{mass}\;(g)}\;\times \;100\%$$

An additional experiment was conducted to estimate the boiling temperature elevation of the water in potatoes. To achieve this, liquid was extracted from almost 2 kg of potatoes, using a juicer (Nutribullet Juicer 800W, Los Angeles, CA, USA), heated and its boiling temperature over time was recorded using a data logger. The change in the mass of the liquid was measured at regular intervals, using an electronic kitchen scale (Wiltshire, NSW, Australia). A correlation was developed to estimate the boiling temperature elevation as a function of potato mass. It should be noted that the data obtained was used to approximate the correlation between the water loss during frying and the boiling temperature elevation since potato liquid does not have the same physicochemical properties as a potato. Furthermore, it was difficult to conduct experiments above 120 °C as the liquid solution became very viscous and starch gelatinization occurred which hindered water evaporation.

## 3. Mathematical Modeling

An approximate quasi-steady state was used to estimate the complete frying time of potato and the frying process modeled by VSN and enthalpy methods using MATLAB software (version R2020b, The MathWorks Inc., Natick, MA, USA).

### 3.1. VSN Method

In the VSN method, it is assumed that there are two regions of the potato discs separated by an interface namely the “core” and “crust” regions [4]. The physical properties of potato for the two regions of core and crust used in this study are summarized in Table 1. The potato disc sample was assumed to be an infinite slab comprised of a porous solid structure filled with water. The effect of shrinkage and water diffusion in the core region were assumed to be minimal as they were fully saturated with water [4].

To determine the temperature of the potato discs over time, the heat conduction equation (Equation (4)) in the form of sensible heat was first solved for the whole potato, explicitly using a finite difference method:

(4) $$\frac{\partial T}{\partial t}=\alpha \;\frac{\partial^{2}T}{\partial y^{2}}\;$$

where *T* and *t* are temperature and time, respectively; *α* is thermal diffusivity of potato and *y* refers to the position from the surface of potato disc.

As soon as the surface temperature of the potato disc reached its boiling point, a thin layer was assumed to form a crust region needed to start the computation with the presence of two regions as required by the VSN method [4]. Following this, Equation (4) was then solved for both crust and core regions using the following initial and boundary conditions (Equations (5)–(9)):

(5) $$T=0\;T=\;T_{i}\;\mathrm{where}\;T_{i}=20\;{}^{\circ}C$$

(6) $$y=0\;h\;(T_{0}\;-\;T_{s})=-k_{cr}\;\frac{\partial T_{s}}{\partial y}\;\mathrm{where}\;T_{0}=180\;{}^{\circ}C$$

(7) $$y=\frac{\delta }{2}\;\frac{\partial T_{\infty }}{\partial y}=0\;$$

(8) $$y=Y_{n}\;T_{co\;}{=T}_{cr}=103\;{}^{\circ}C$$

(9) $$y\;=\;Y_{n}\;\rho_{co}{\;\lambda }_{w}\;w\;\frac{{dY}_{n}}{dt}=k_{cr}\;\frac{\partial T_{cr}}{\partial y}-\;k_{co}\;\frac{\partial T_{co}}{\partial y}\;$$

where subscripts “*s*”, “*cr*” and “*co*” represent the surface, crust, and core of potato disc, respectively. Subscript “*i*” refers to initial condition of potato and “*o*” refers to oil. *δ* denotes the full thickness of potato disc. *w* and *λ_w_* define the moisture content, and latent heat of boiling of water, respectively. *Y_n_* represents the interface position at each timestep, and *h* and *k* are convective heat transfer coefficient and thermal conductivity of the potato, respectively.

The two regions were then divided into equal space increments. To start the computation, a linear temperature distribution was assumed within the initial crust layer. The magnitude of error raised by this simplification was negligible if the assumed crust thickness was small. An extremely small initial crust layer should be avoided; otherwise, computation time would increase substantially to satisfy the stability requirement of the explicit finite difference method. An initial crust layer of between 1% and 2.5% of the potato disc thickness was deemed to be sufficient [4].

As frying proceeds, the crust thickness will increase and hence the space increment width will expand, while the space increment width of core region will decrease. Using the explicit finite difference formulation described by Murray and Landis [11], the nodal equations for both regions are formulated and used to calculate the dimensionless temperature. Equations (10) and (11) represent the nodal discretization of crust and core regions, respectively.

(10) $$\theta_{j,n+1}{=\theta }_{j,n}+j\left(\theta_{j+1,n}\;-\;\theta_{j-1,n}\right)\;\times \;\frac{\left(Y_{n+1}\;{-\;Y}_{n}\right)}{{2Y}_{n}}{+\alpha }_{cr}∆{tJ}^{2}(\theta_{j-1,n}\;-\;2\theta_{j,n}+\theta_{j+1,n})Y_{n}^{2}\;$$

(11) $$\theta_{j,n+1}=\;\theta_{j,n}+\left(2J\;-\;j\right)\left(\theta_{j+1,n}\;-\;\theta_{j-1,n}\right)\;\times \;\frac{Y_{n+1\;}-\;Y_{n}}{\delta \;-\;Y_{n}}+\;\alpha_{co}∆{tJ}^{2}{(\theta }_{j-1,n\;}{-\;2\theta }_{j,n\;}+\;\theta_{j+1,n})(\delta \;-\;Y_{n}{)}^{2}\;$$

where *θ* is the difference between temperature and the water boiling temperature (*T* − *T_b_)* and subscripts “*j*” and “*n*” are space and time increments, respectively. *J* represents the number of spatial increments in the crust and core regions. The interface position may be calculated from Equation (12).

(12) $$Y_{n+1\;}{=Y}_{n\;}+\left\{\frac{∆t}{\rho_{co}{\;w\;\lambda }_{w}}\right\}\;\times \;[\left(\frac{0.5{\;k}_{cr\;}J}{Y_{n}}\right)\left(\theta_{J-1,n}\;{-\;4\theta }_{J,n}\right)+\frac{0.5{\;k}_{co}\;J\left(\theta_{J+3,n}\;{-\;4\theta }_{J+2,n}\right)}{{\delta \;-\;Y}_{n}}]$$

During the two-region period (when both core and crust are present), heat is assumed to transfer from the hot oil to the potato disc surface through a convection boiling process with a coefficient higher than the convection coefficient used for a single region of core or crust, in this case 500 versus 250 W/m^2^∙K (Table 1) [4]. As the interface position approaches the center of the potato disc (i.e., a distance equivalent to 1% to 2.5% of potato disc thickness [4]), the two region computation changes to a single region computation as only the crust is being considered. The sensible heating of the crust region was calculated using Equation (4).

### 3.2. Enthalpy Method

In the enthalpy method, water evaporation is assumed to occur over a narrow arbitrary region of boiling (*T_b_* ± *ε*) °C, where *ε* is a small value (in the order of 0.5 °C) representing half the phase change temperature [28]. Since a potato disc was assumed to be an infinite slab [4], the assumption of one-dimensional heat transfer became valid. This assumption meant that Equation (13) could be solved using initial and boundary conditions defined by Equations (5)–(7), using an explicit finite difference method (detailed explanation referred to the study of Gholamibozanjani and Farid [29] for modeling melting and solidification). To guarantee stability of the finite difference method, the condition set by Equation (15) need to be met.

(13) $$\rho \frac{\partial H}{\partial t}=k\;\frac{\partial^{2}T}{\partial y^{2}}\;$$

where *H* is enthalpy as described by Equation (14).

(14) $$H=\{\begin{array}{cc} C_{co}T, & T\;<\;T_{b\;-\;\varepsilon }\\ \left(T\;-\;\left(T_{b\;}-\;\varepsilon \right)\right)\;\times \;\left(\frac{C_{co}{+C}_{cr}}{2}+\frac{{w\;\lambda }_{w}}{2\;\varepsilon }\right){+C}_{co\;}\times \;\left(T_{b}\;-\;\varepsilon \right), & T_{b}\;{-\;\varepsilon \;<\;T\;<\;T}_{b}+\varepsilon \\ C_{cr}{\;T+w\;\lambda }_{w}+\left(C_{co}\;{-\;C}_{cr}\right){\;\times \;T}_{b}, & T\;>\;T_{b}+\varepsilon \end{array}$$

(15) $$\alpha \frac{∆t}{∆y^{2}}\leq \frac{1}{2}\;$$

where subscript “*b*” denotes the water boiling temperature.

In this single-phase method, computation is conducted for three periods: First the sensible heating of the potato until its core temperature reaches the lower bound of the evaporating point of water, (*T_b_* − *ε*) °C. During this sensible heating period, the specific heat capacity and other physical properties of the potato are assumed to be constant in the core region (Table 1). Once the potato temperature reaches (*T_b_* − *ε*) °C, it is assumed that a two-phase region (liquid and vapor) is formed, and evaporation starts. During this water evaporating period, between (*T_b_* − *ε*) °C and (*T_b_* + *ε*) °C, enthalpy rises dramatically. Finally, the sensible heating of the potato crust starts when the potato temperature reaches (*T_b_* + *ε*) °C.

### 3.3. Approximate Quasi-Steady State

Farid et al. [23] developed an analytical method to calculate the approximate time required for complete frying as defined by Equations (16) and (17):

(16) $$\beta =\frac{\left(T_{o}\;-\;T_{b}\right)}{\rho \;\lambda_{w}\;\varepsilon }$$

(17) $$t=\frac{1}{\beta }\;(\frac{\delta }{2\;h}+\frac{\delta^{2}}{8\;k_{cr}})$$

Equation (17) is derived by ignoring sensible heat, based on the assumption that the temperature reaches the boiling point of water very fast, compared to the time required to complete frying. This sensible heat is considered to be negligible compared to the high latent heat of water vaporization since it accounts for less than 2% of heat absorbed during frying [30].

## 4. Results and Discussion

### 4.1. Experimental Measurements

#### 4.1.1. Effect of PEF Pre-Treatment on the Moisture Content, Thermal Conductivity and the Temperature-Time Profile of Potatoes during Frying

The average moisture content of the potatoes (considering both the surface and center) in the absence of PEF treatment was 73.5 wt %. As PEF Low- and PEF High treated potatoes a moisture content of 74.4 wt % and 73.3 wt %, respectively, this data shows that that PEF treatment had little effect on the moisture content of the potatoes (Table 2). The results also showed negligible difference for moisture content within the potato from surface to center based on the measurement technique used in this current study.

The thermal conductivity of both untreated PEF treated potatoes was estimated before and after frying and the values obtained were used in Equations (4), (10)–(13), (15) and (17) and the boundary conditions were calculated from Equations (6) and (9). The predicted thermal conductivities of untreated and PEF-treated potatoes are shown in Table 3. It was found that thermal conductivity of fried discs from untreated and PEF-treated potato tubers were in the range between 0.10 and 0.11 W/m∙K. The average value for the thermal conductivity of fresh potatoes (without frying) was 0.73 W/m∙K, with a variation from 0.68 to 0.78 W/m∙K. Hence, PEF treatment was concluded not to have a clear impact on thermal conductivity of potato (Table 3). The water content in food is known to be a key factor influencing its thermophysical properties [31,32]. Therefore, negligible difference in the thermal conductivity for untreated and PEF-treated potatoes is expected as they had a similar moisture content (Table 2). Note that the average values of thermal conductivity (0.73 W/m∙K for fresh potato and 0.11 W/m∙K for fried potato) were used as the basis of modeling in Section 4.2.

**Figure 4:**
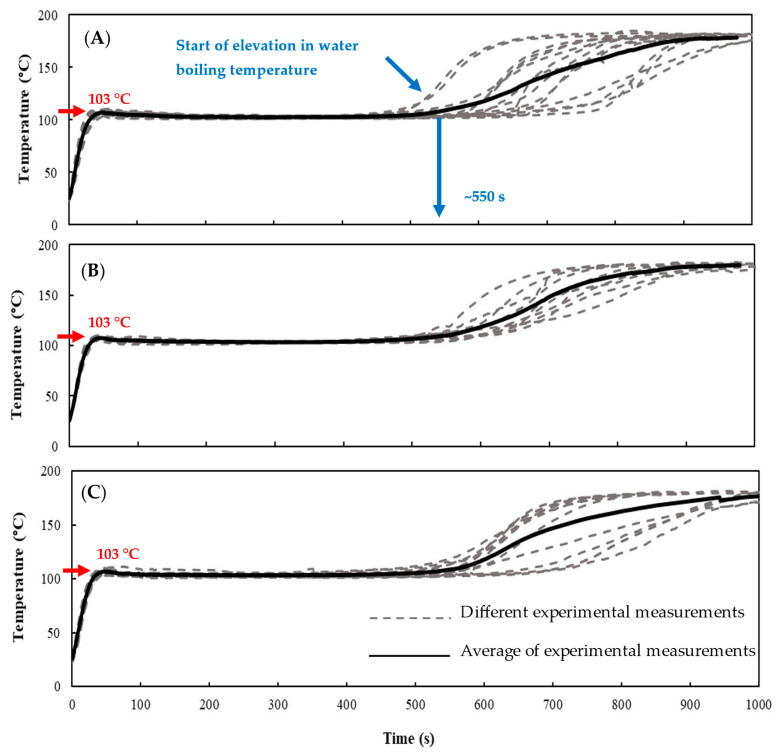
Experimental measurements of center temperature of potato discs fried at 180 °C for (**A**) untreated, or PEF-treated at (**B**) PEF Low (1.1 kV/cm, 54.8–57.7 kJ/kg) or (**C**) PEF High (1.1 kV/cm, 149.2–159.6 kJ/kg), where water in the potato discs typically boiled at 103 °C (red arrows) and starts rising after around 550 s (blue arrows). Gray dash lines in (**A**–**C**) represent experimental measurements from 10 independent potatoes. Black solid lines in (**A**–**C**) show the average measurements (*n* = 10 independent potatoes).

The temperature-time profiles of untreated and PEF-treated potatoes during frying were obtained when the 5 mm-thick potato disc samples were fried in an oil bath at 180 °C (as described in Section 2.4). The measured center temperature for both untreated and PEF treated potato discs are depicted in Figure 4, where different experimental measurements are shown in gray dashed lines and the average of all measurements is shown with a black solid line. The average center temperature of the potato disc for the untreated and PEF-treated potatoes shared very similar temperature-time profiles.

As frying proceeded, the center temperature of the potato experienced an initial sharp ascending (sensible heating) period, followed by a leveling off at around 103 °C (Figure 4, see red arrows) (evaporation), and a final moderate ascending period (sensible). In the initial period, the water temperature of the potato increased sensibly to its boiling point and then stayed constant at around 103 °C for an extended period. All experimental values measured in the current study as well as those reported by other researchers consistently show that the initial boiling temperature of water in potatoes is approximately 103 °C [2,4,33,34]. The elevation of the boiling point is believed to occur as a result of nucleation of steam bubbles in the superheated interface separating crust and core [34]. Therefore, the initial boiling temperature of water (*T_b_*), in both untreated and PEF-treated potatoes, was set to 103 °C, throughout the following modeling section (in Equations (14), (16) and (21)).

As the interface moves close to the center of the potato, the water inside the potato was concentrated with soluble matters such as sugar and potassium, leading to an elevation in water boiling temperature [35,36]. In this study, the elevation of water boiling temperature was observed to occur at around frying time of 550 s (Figure 4, see blue arrows) and then the average center temperature of the potato discs rose steadily after about 550 s.

**Figure 5:**
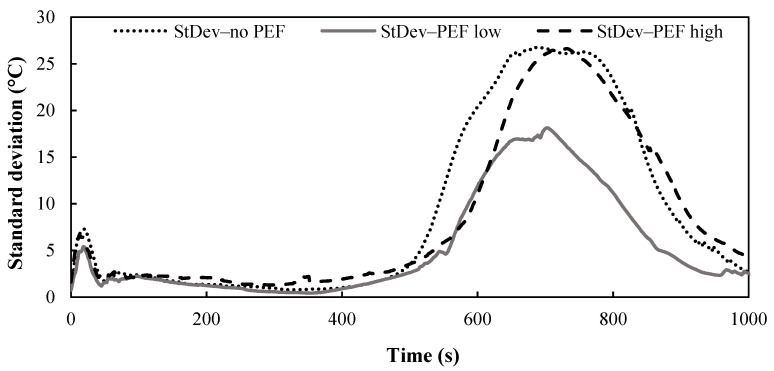
Averaged standard deviation of the measured center temperature for untreated and PEF treated potato discs (*n* = 10 independent potatoes for each treatment).

In the current study, a huge variation in the temperature-time profiles between individual potato discs occurred, probably due to the biological difference between the (untreated) potatoes. Figure 5 illustrates the extent of variation in the center temperature as a function of frying time for untreated and PEF-treated potatoes. During the first 500 s of frying, all the potato disc samples, regardless of pre-treatment, experienced similar temperature-time profiles from the start of the frying until the center temperature of the disc reached the water boiling temperature. This was illustrated by the low standard deviations obtained for the data over the first 500 s of frying where the center temperatures of different potato discs were situated very close to the average value (Figure 5). As the frying lapsed after 550 s, and the center temperature of the disc rose above the water boiling temperature until it reached the oil temperature, a considerable variation in the experimental measurements between individual potatoes was observed. At this stage, the standard deviations of the experimental temperature measurements between individual potatoes were high, where the center temperature values spread out over a wider range, of 26, 18 and 26 °C above the boiling temperature for untreated, PEF Low treated and PEF High treated potatoes, respectively (Figure 5). It is important to note that the standard deviation of the measured center temperature for different potato discs showed that the PEF Low treatment has reduced the experimental variation, by up to 30% compared to untreated potatoes and those pre-treated with PEF High. Potatoes pre-treated with PEF Low had a lower variation in their temperature-time profiles than previously reported in the literature. This result could be because PEF causes loss of cell compartmentalization and allows water to be redistributed across various cell compartments in plant tissue [37], including extracellular spaces [38]. Changes in the water distribution within potato cells would likely influence the water evaporation process from potato discs during frying. In contrast, the high standard deviation in the center temperature measurements of potato discs pre-treated under PEF High may have occurred because the high intensity treatment had severely disrupted the potato cell, ultimately leading to the collapsed of the cell walls [24,39,40].

#### 4.1.2. Increase in the Boiling Temperature of Water in Potato during Frying

The potato discs were removed from the fryer (at 180 °C) when the center temperature exceeded 103 °C and the percentage of water remaining in them was estimated (Table 4). Approximately 72% to 86 % of the water in potato was removed during the initial frying period.

**Figure 6:**
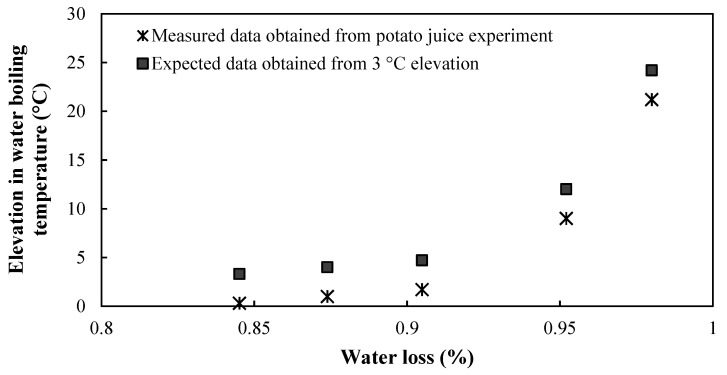
Elevation in water boiling temperature achieved from boiling the untreated potato liquid (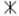) and 3 °C temperature correction expected (■) due to the effect of steam bubble nucleation in the superheated interface separating crust and core.

Following the potato liquid boiling experiment described in Section 2.5, an exponential trend between water boiling temperature elevation and the water loss percentage was found as water was removed due to evaporation (Figure 6). The obtained curve was shifted along the temperature elevation axis by 3 °C to compensate for the nucleation of steam bubbles in the superheated interface separating crust and core which occurs during frying (but not in the potato liquid). It was then extrapolated to achieve a 100 wt % water loss, as it was difficult to obtain from the experiment explained in Section 2.5.

Based on the results reported in Table 4, the experimental measurements of the potato liquid boiling temperature (measured data in Figure 6) and data extrapolation to 100 wt % water losses, Equation (18) was developed. It was assumed that the location of the boiling interface determined the water remaining in the potato disc during frying and hence its boiling point.

(18) $$∆T_{b}{=7\;\times \;10}^{-10}\;\exp(25.455\;\times \;\frac{Y_{m}}{\delta /2})$$

It has to be noted that Equation (18) is an oversimplification of the frying process of potato discs. During frying, not only water evaporates that causes an increase in the soluble sugar and salt concentration, but the potatoes also experience reactions such as starch gelatinization and protein denaturation [41] which have not been considered in this study. Then, the fixed water boiling temperature (*T_b_*) in Equations (10)–(12) and Equations (13) and (14) were replaced with the variable boiling temperature of Equation (18) to be solved via the VSN and enthalpy methods, respectively.

### 4.2. Mathematical Modeling Results

**Figure 7:**
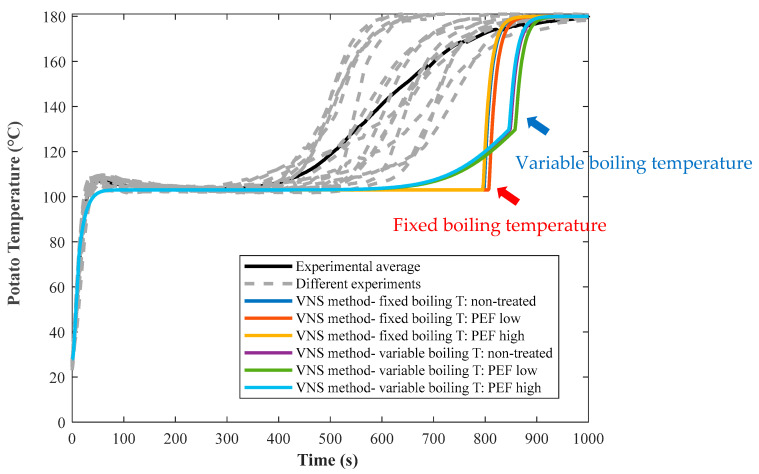
Model prediction (colored lines) of measured center temperature for untreated, PEF Low and PEF High treated potato discs (5 mm thickness) fried at 180 °C, using VSN method, considering at fixed (those indicated with red arrows) and variable (those indicated with blue arrows) boiling temperatures of the water in the discs.

In the current study, the frying process of untreated and PEF-treated potatoes was modeled following three methods (VSN, enthalpy and approximate quasi-steady state) as described in Section 3. These models were applied for two conditions, namely: (i) at the constant boiling temperature of water in potato at 103 °C (fixed conditions, based on the results in Figure 4) and (ii) when the boiling temperature of water in the potato increases (variable conditions) following Equation (18). Figure 7 shows the application of the VSN method using a fixed and variable water boiling temperature to predict the measured center temperature of untreated and PEF-treated potato discs. In the VSN model, as the interface position moves towards center, the space increment width in the core side becomes very small, requiring a very small time-step that otherwise would create numerical instability. To overcome this computational problem, when the space increment reached a distance about 2% of the potato thickness from the center, the computation was carried out based on a single phase of crust region. As a result, a jump in the center temperature of the potato disc was observed as shown in Figure 7 (indicated by red and blue arrows when fixed and variable boiling temperatures were used, respectively).

The modeling results were validated against the experimental measurements of untreated, PEF Low and PEF High-treated potatoes considering the average values of moisture content and thermal conductivity reported in Section 4.1.1 (Table 2 and Table 3 respectively).

The measured center temperature of the potato disc was also predicted using the enthalpy method described in Section 3.2. In this method, each location went through three regions namely core, mushy (mixture of core and crust) and crust. Thermal diffusivity for core and crust regions was calculated using Equation (19):

(19) $$\alpha_{cr}=\frac{k_{cr}}{\rho_{cr}\;C_{cr}}\;\alpha_{co}=\frac{k_{co}}{\rho_{co}\;C_{co}}\;$$

In the mushy phase, the density of the crust and core was defined according to Equation (20) [29]. In fact, as frying proceeds, oil penetrates into the potato disc making it difficult to measure its density and hence its thermal diffusivity.

(20) $$\rho_{\mathrm{mushy}}=\left(1\;-\;X_{n,m}\right)\rho_{co}+X_{n,m}\rho_{cr}\;$$

where *X* denotes the crust fraction of potato and is calculated from Equation (21).

(21) $$X_{n,m}=\;\frac{T_{n,m}\;-\;(T_{b}\;-\;\varepsilon )}{2\;\varepsilon }$$

where subscript “*mushy*” refers to the phase where a mixture of core and crust exists.

**Figure 8:**
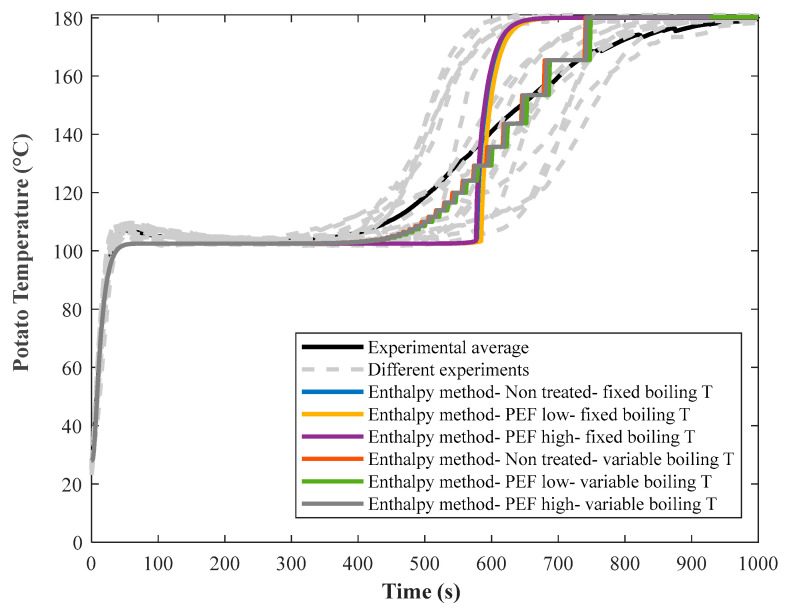
Model prediction (colored lines) of measured center temperature for untreated, PEF Low and PEF High treated potato discs (5 mm thickness) fried at 180 °C, using enthalpy method and the density of the mushy region (Equation (17)), considering fixed and variable boiling temperatures of the water in the discs.

Figure 8 illustrates the model prediction based on the experimental measurements of 5 mm-thick untreated, PEF Low and PEF High treated potato discs fried at 180 °C, using the enthalpy method under the conditions of fixed and variable water boiling temperature. Unlike previously developed models [2,4], the current model predicted the experimental temperature-time measurements of potatoes reasonably well, especially at the final stage of frying. Grid and time-step independence analysis was also assured for the enthalpy method following the approach explained in a study conducted by Gholamibozanjani and Farid [29].

**Figure 9:**
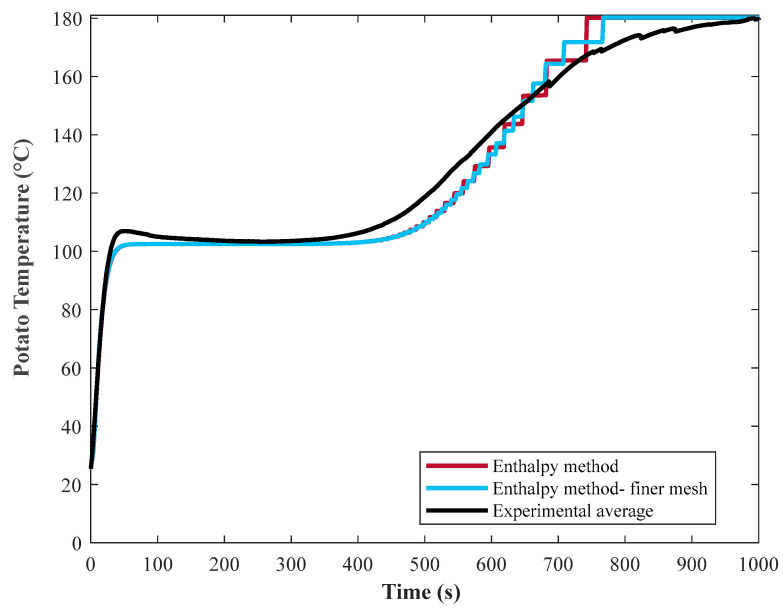
Effect of more fine mesh size during the computation of enthalpy method on the smoothness of graph using untreated temperature-time profile as an example.

By increasing the space increments and applying smaller mesh sizes during computation, a smoother graph was achieved as shown in Figure 9. In general, increasing the space increments does not contradict the grid independency analysis [29], as the latter verifies the stability of the numerical solution, whereas increasing space increments makes the profile smoother.

**Figure 10:**
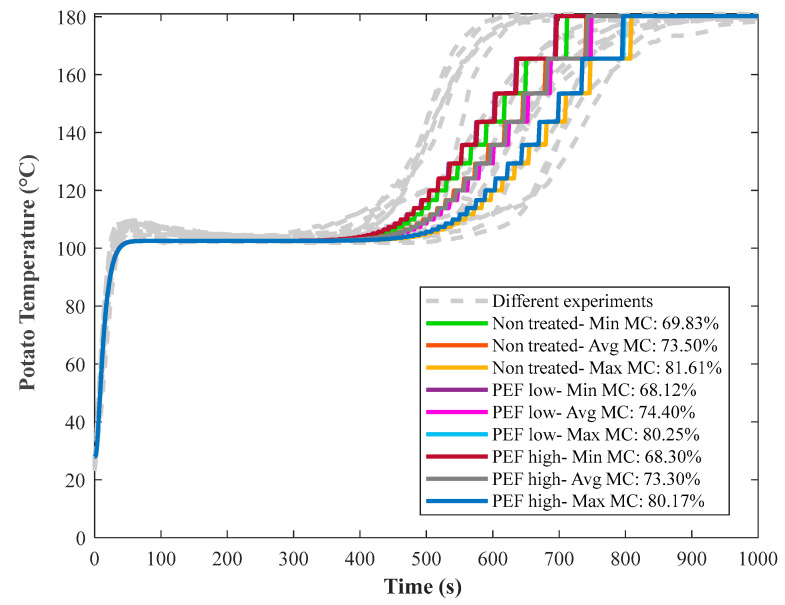
Model prediction (colored lines) of center temperature for untreated, PEF Low and PEF High treated potato discs (5 mm thickness) according to their minimum, average and maximum measured moisture contents (MC) (see Table 2), when fried at 180 °C.

Moisture content is an important factor in determining the temperature-time profile of a potato disc. Figure 10 shows the model prediction of the temperature of potatoes based on the minimum, average and maximum measured moisture contents, at both the surface and center of the potatoes, of 69 wt %, 73.8 wt % and 80 wt % for the untreated, PEF Low and PEF High treated potatoes, respectively (Table 2). Along with the effect of other phenomena occurring during the frying process of potato such as gelatinization and PEF pretreatment, the difference in the potato moisture content explains the variation in experimental measurements as shown in Figure 10.

The advantage of the enthalpy method compared to the VSN method is that the former does not face any computational problems and it can be applied to multi-dimensional geometry samples, which is not the case with the VSN method. The approximate quasi-steady state method (Equation (17)) of Smith and Farid [23] which considers core density and crust thermal conductivity as the basis of calculations, shows that the complete frying of a 5 mm-thick potato disc happens after 809 s, which is compatible with those obtained from VSN and enthalpy methods based on keeping the water boiling temperature constant.

## 5. Conclusions

The developed model, which integrated a variable water boiling temperature into the conduction heat transfer equation and was solved by the enthalpy method, predicted the experimental measurements during potato frying reasonably well. The VSN method suffers from numerical instability when the explicit finite difference is used in its solution. This limitation was eliminated via the use of enthalpy method. The enthalpy and the Variable Space Network (VSN) methods based on a moving interface defined by the boiling temperature of water in potato during frying could adequately describe the temperature time distribution of untreated and PEF-treated potatoes even though PEF treatment did not dramatically change the initial moisture content, thermal conductivity or the total frying time for the potatoes to reach the targeted oil temperature. It was pleasing to note that PEF treatment reduced the variations in the experimental measurements of the potato center temperature time profile by up to 30%, when a low PEF treatment intensity (1.1 kV/cm and 54.8–57.7 kJ/kg) was used. This finding implies that it will narrow the confidence interval between the predicted and measured temperature time distribution. As a consequence, the process uniformity of PEF processing can be more controlled and the changes in quality attributes of potato discs during frying, such as brown color formation and crispiness, can be predicted more accurately.

## Figures and Tables

**Table 1:** Physical properties of potato discs.

Property	Core	Crust	Reference
Thermal conductivity of potato, *k* (W/m∙K)	0.73	0.11	Current work
Potato density, *ρ* (kg/m^3^)	1132	386	[4]
Specific heat capacity of potato, *C_p_* (kJ/kg∙K)	3.450	1.790	[4]
Parameter	Value		
Convection heat transfer coefficient	250 W/m^2^∙K		[4]
Boiling heat transfer coefficient	500 W/m^2^∙K		[4]
Initial moisture content	0.738		Current work

**Table 2:** Average, minimum and maximum moisture content (wt %) of the surface and center of PEF-treated and untreated potatoes before frying.

	Potato Surface			Potato Center			Overall Average ^†^
	Average *	Min	Max	Average *	Min	Max	
Untreated	72.7± 2.8	69.8	76.9	74.3 ± 3.3	70.1	81.6	73.5
PEF Low	75.6 ± 4.2	71.2	80.2	73.3 ± 3.1	68.1	76.0	74.4
PEF High	73.6 ± 4.7	68.6	80.2	73.1 ± 3.8	68.3	79.0	73.3

* Data presented as average ± standard deviation from 5 independent potato tubers (*n* = 5). ^†^ Overall average considering the potato surface and potato center for each pre-treatment applied to the potatoes.

**Table 3:** Average, minimum and maximum thermal conductivity (W/m∙K) of fresh and fried potatoes.

	Fresh Potato			Fried Potato		
	Average *	Min	Max	Average *	Min	Max
Untreated	0.73 ± 0.04	0.68	0.75	0.11 ± 0.01	0.10	0.11
PEF Low	0.73 ± 0.03	0.71	0.76	0.11 ± 0.01	0.10	0.11
PEF High	0.78 ± 0.01	0.77	0.78	0.10 ± 0.00	0.10	0.10

* Data presented as average ± standard deviation from 5 independent potato tubers (*n* = 5).

**Table 4:** Average, minimum and maximum percentage of water remained in partially fried potatoes when the center temperature exceeded 103 °C.

Experimental Measurements	Average *	Min	Max
Initial potato mass before drying (g)	6.4 ± 0.9	5.6	7.5
Initial water mass (g) ^a^	4.8 ± 0.7	4.0	5.6
Water remained after partial frying (g) ^b^	1.0 ± 0.3	0.6	1.4
% of water remaining after partial frying ^c^	21.0 ± 5.3	14.0	28.0
% of water removed after partial frying ^d^	79.0 ± 5.4	71.5	85.6

* Data presented as average ± standard deviation from 5 independent potato tubers (n = 5). ^a^ Calculated based on Equation (1). ^b^ Calculated based on Equation (2). ^c^ Calculated based on Equation (3). ^d^ Percentage of water removed after partial frying = 100% − percentage of water remaining after partial frying.

## Abbreviations

Nomenclature	
*C*	specific heat capacity (J/kg∙K)
*H*	convection/boiling heat transfer coefficient (W/m^2^∙K)
*H*	enthalpy (J/kg)
*k*	thermal conductivity of potato (W/m∙K)
*t*	time (s)
*J*	number of spatial increments in the crust and core regions
*T*	temperature (°C)
*w*	moisture content (dimensionless)
*X*	crust fraction (dimensionless)
*y*	distance from the surface of potato disc sample (m)
*Y*	distance of the interface from surface of potato disc sample which separates the core and crust regions (m)
Greek symbols	
*α*	thermal diffusivity of potato (m^2^/s)
*δ*	full thickness of the potato disc (m)
*ε*	an arbitrary value representing half the phase change temperature (°C)
*θ*	difference between temperature and the water boiling temperature (°C)
Δ	increment
*ρ*	density (kg/m^3^)
*λ_w_*	latent heat of boiling of water (J/kg)
Subscripts	
*b*	boiling
*co*	core
*cr*	crust
*i*	initial state
*j*	space increment
*n*	time increment
*mushy*	a region with a mixture of core and crust
*o*	oil
*p*	potato
*s*	surface of the potato disc sample
